# A model of an agile organization designed to better manage the COVID-19 crisis

**DOI:** 10.1177/0840470420980478

**Published:** 2020-12-23

**Authors:** Fabrice Brunet, Kathy Malas, Danielle Fleury

**Affiliations:** 1 Executive Office, 25443 Centre hospitalier de l’Université de Montréal, Montreal, Quebec, Canada.; 2 Research Centre, 25443 Centre hospitalier de l’Université de Montréal, Montreal, Quebec, Canada.

## Abstract

COVID-19 strongly hit healthcare organizations due to three factors: the lack of knowledge of this new virus, the fear of the people, and the continuous modifications in the management of the crisis. This situation required flexibility and adaptability of organizations, as our university health centre demonstrated. It relied on a decentralized model of management based on three pillars: a culture of innovation and creativity, an agile organizational structure, and an open innovation ecosystem and network. These assets were already developed prior to the onset of COVID-19 and helped our organization to better respond to the crisis.

## Introduction

All healthcare systems and organizations around the world were strongly hit by COVID-19. Their response depended on their agility and capacity to adapt to the magnitude and severity of this new and infectious disease. This crisis was unprecedented in its rapid spread and its economic, political, and social impact, which directly and indirectly affected both healthcare workers and the general population.

We want to share how our university health centre in Canada enhanced its response to the crisis using an organic enterprise model.^1^

## Three pillars in developing an organic enterprise prior to COVID

**Figure 1.**
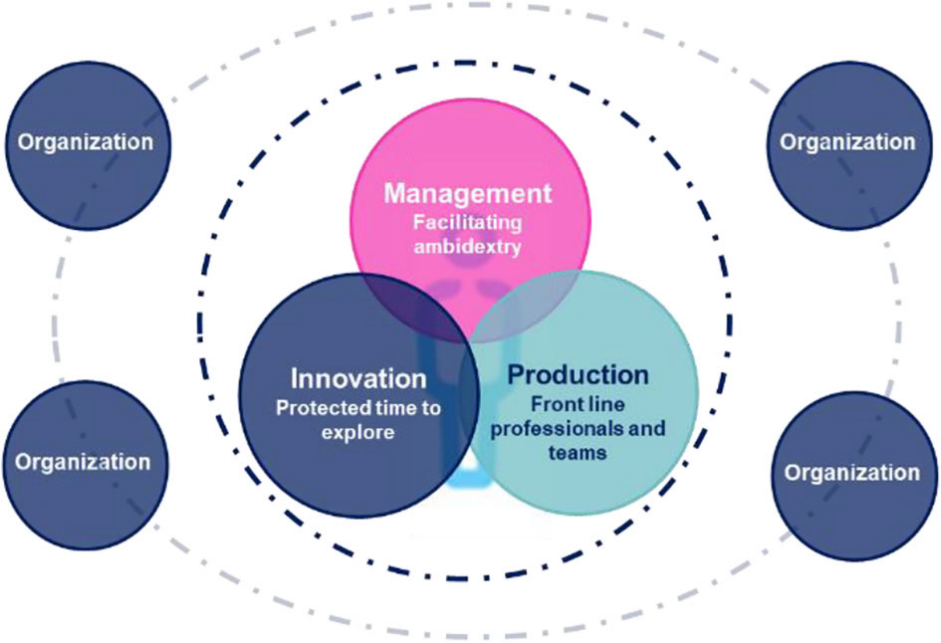
CHUM’s organic model. CHUM indicates Centre hospitalier de l’Université de Montréal.

Over the last 5 years, the Centre hospitalier de l’Université de Montréal (CHUM) has developed and implemented a new organizational model to enhance its adaptability and agility when responding to successive crises, following the amalgamation of three old hospitals into a new one. Before the onset of the pandemic, CHUM had introduced a new vision of a dynamic organization which would harness innovation and creativity to better respond to the needs and demands of the population, using a model of organic enterprise as described by Mintzberg.^1^ This model is based on three pillars: (1) a culture of creativity and innovation supported by leadership and “communityship” (2) an agile organizational structure and mechanisms, and (3) an open innovation ecosystem and network (Figure 1).

The culture of creativity and innovation was an essential pillar in our transformation. Every individual and team contributed their experience, knowledge, and expertise to solve problems in their local context and thus to better respond to the needs of patients and teams. Recognizing these different areas of expertise and talent was also an essential component of the culture. The CHUM developed a talent management office to enable every individual to develop their own skills and career pathways according to their personal and professional goals. Furthermore, offering individuals and teams time not only to produce and deliver care but also to explore encourages them to be inventive and consider new solutions.

This culture was promoted by a vision-oriented and reason-focused leadership: a consistent emphasis on innovation by local teams^2^ to constantly improve the health and well-being of people and the performance of the organization. Managers of all levels were in close communication with frontline workers and teams, clearly expressing the overall vision and attending to their needs and problems, to make them feel empowered. Patient partnership are a fundamental component of our mission and they are part of our interdisciplinary teams. The CHUM in collaboration with the Centre of Excellence on Partnership with Patients and the Public have more than 3,000 patients and citizen partners who collaborate in different innovative projects in care, research, education and strategic committees.

Leadership was complemented by a strong sense of community within the organization. “Communityship” is achieved when every individual exercises leadership and partnership and works together towards a set of common goals.^3,4^ This “communityship” was nourished by both informal and formal activities, such as communities of innovation, brainstorming sessions, and cooperative competitions, which amplified creativity and strengthened commitment.

The innovation of individuals and teams is also essential to our second pillar, which proposes an ambidextrous and agile organizational structure.^5,6^ The CHUM implemented such an organization and capitalized on top-down, bottom-up and transversal mechanisms.^7^ It assigned expert innovators to the task, aided by a transversal pole of innovation and Artificial Intelligence (AI) in health, which enhanced the expertise of multiple teams.

Furthermore, coordination mechanisms were applied to assist exploration and production functions, making sure relevant and impactful solutions were introduced into care and services. Communities of practices within the organization promoted knowledge sharing, which accelerated learning within and between multiple departments. Another example of such a coordination mechanism is the innovation cycle. This process takes ideas or problems and validates their relevance while codeveloping, experimenting, implementing, assessing their value and impact, and adopting them in practices. This cycle promotes creativity, structures and accelerates innovation, and capitalizes on learning. Also, measuring impact and value for patients, teams, and the organization is key in this process. At CHUM, we deployed a systemic approach to measuring value, based on the quadruple aim in health.^8,9^ To these four aims, CHUM adds knowledge generation and mobilization in fulfilling its academic mission.

The third and final pillar is the open innovation ecosystem, which amplifies creativity and innovation of individuals, teams, and the organization.^10^ The CHUM has put in place several knowledge and innovation networks, both locally and internationally, that share experiences and collaborate on projects. Within its organizational processes and practices, it embedded the interdisciplinary and intersectoral partnership from the project’s conception. This is translated by working in an open mode from the need assessment or ideation and includes patients, healthcare professionals, researchers, private industry partners, universities, and decision-makers.

This previously described transformation allowed CHUM to cope with multiple internal and external crises, such as the amalgamation of three hospitals into one, the Ebola crisis, and the implementation of AI in practices. This culture, combined with an agile structure, also prepared the teams to quickly and appropriately respond to the new sanitary crisis.

## Crisis management: Three factors countered by three organizational pillars

Three factors in the COVID-19 crisis made its management complex^11^ and influenced its global response. Firstly, the disease was unknown and lacked standard of care. The second factor was how vastly and rapidly the situation changed, both locally and at government level, which increased its complexity and uncertainty. And finally, COVID-19 is a life-threatening disease, which generated a high level of fear from patients and healthcare workers.

The COVID-19 crisis can be compared to trauma.^12^ Coping mechanisms developed prior to a trauma can help an individual, team, or organization respond to it. Hence, CHUM relied on the three pillars established in its previous transformation, as described above, to function as a response mechanism in fighting this crisis.

To counter the first factor—the unknown nature of the disease and the lack of pre-existing practices—real-time and rapid integration of new knowledge, training, and experience generated by experimentation and research was encouraged at all levels of the organization. For example, the CEO and senior management studied and followed international COVID-19 trends. Frontline clinicians and employees, researchers, and experts in pedagogy worked together to develop new protocols with known and unknown elements, testing them rapidly with techniques such as simulation and diffusing them at large to more than 7,500 healthcare professionals within the hospital and other institutions. New research protocols (more than 70), such as an image bank and biobank with COVID-19 patients, were rapidly created to generate new answers to unknown questions. Thus, the culture of innovation helped multiple teams in mobilizing real-time knowledge to counter the unknown. Frequent communications were used to keep frontline workers and patients constantly informed of new information.

As per the second factor, we mitigated the constant daily changes by adopting an agile structure and managerial mechanisms to respond rapidly. A crisis command centre was created with senior management. Its role was to mitigate these rapid changes in partnership with the CHUM community. In addition to this, subcommittees were formed to pass from strategic decision-making to operations, and vice versa. A special tactic team was also introduced to quickly identify needs and problems faced by local teams and address them to the command centre. Local teams were invited to the command centre to present their innovative practices. Teams promptly experimented and implemented multiple solutions using an interdisciplinary and intersectoral approach of monitoring indicators of quality, security, and patient experience. This adaptability was possible in a time of crisis due to the agile organization and the coping and response mechanisms developed prior to COVID-19. The teams felt a sense of autonomy in decision-making and were able to connect with different teams, the crisis command centre, and other subcommittees when needed.

The third pillar in our previous transformation is the open and inclusive innovation ecosystem. An example of such initiative resulting from our ecosystem is the on-line community of 3D printing and engineering. This community was created and deployed in 3 days and included researchers, engineers, clinicians, citizens, managers, procurement experts, and private industry. They quickly identified the needs and problems of clinicians and teams and tested and developed 3D solutions to shortages of personal protective equipment and materials, such as 600 face shields printed per week and COVID-19 swab testing. Another example is the opening of a previously closed hospital for COVID and non-COVID patients who were stable, yet incapable of returning to their long-term facilities as they were still infectious. In less than a week, CHUM, with the help of teams, volunteers, and other healthcare professionals, opened a 180-bed hospital. More than 600 clinicians, hygiene and sanitation staff, and other employees were recruited to support patients from the entire Montreal healthcare network. This wouldn’t have been possible without the culture of open creativity and innovation and the structure, processes, and methods that already existed within our organization.

Finally, to counter the last factor of fear from patients and healthcare workers, leadership—from senior leaders to frontline managers—made sure to constantly communicate transparently and authentically to all frontline workers and the CHUM community. Different communication strategies were utilized, such as weekly live webinars with the CEO, weekly meetings with unions, and a web platform for all frontline managers and doctors to diffuse information and answer questions in real time. Furthermore, supported by a research project, we deployed a mobile application where employees could self-screen their level of stress and anxiety. This app also informed employees of support services, such as the COVID psychological phone line. We offered mental health prevention and intervention services for all employees and teams that felt high levels of stress and anxiety. We also helped to tackle the fear and stress of patients and families. Furthermore, a COVID web platform and 24/7 phone line was created for patients, and more than 4,800 patients benefited from such services since March 15, 2020. A dedicated team of nurses and trained employees was created and deployed to answer families’ questions and accompany them in end-of-life visits. More than 130 telephone support and links with clinical teams were made, and 98 visits were made.

## Lessons and perspectives for the second wave

After the first wave, we learned three essential lessons. The first is that the healthcare system can mobilize rapidly by enabling and empowering people on the frontline. The speed of our response, as well as the quality of care and safety measures, was made possible by the collective commitment of our teams and society as a whole. Some barriers, such as regulatory and policy, were lifted, thus accelerating innovation—for example, telehealth consultations at CHUM passed from 700 in mid March to 14,000 in October 2020 after COVID.

The second lesson was the importance of pre-existing coping mechanisms to fight crisis, which strengthened resilience. Resilience of people, teams, and the organization was one of the major observations during this pandemic, which enabled rapid response and recovery. This was possible thanks to patients and citizens, academic, philanthropic and industry partners, frontline workers and managers, who were all driven by vision and motivation to offer the best and most secure care for patients and employees. This commitment, sense of belonging to a community, and ecosystem mobilization were made possible by our organic organization, and the frontline workers and teams finding their own local solutions. They were trusted and listened to by senior and frontline management. Two research projects are ongoing to document and measure how our teams and organization were agile and resilient during this COVID-19 crisis, enabling them to adapt rapidly to such context.^13,14^ As per patients and citizens partnership involvement, we are conducting a research project to measure the impact of nine technological and non-technological innovation deployed during COVID-19 on quality and security of care and patient partnership.^15^ From those projects, one involved the deployment of patient navigators for patients requiring breast cancer surgery (89 interventions of patient navigators for 56 breast cancer patients) and another one is the citizen and volunteer support line to break isolation for COVID and non-COVID patients (more than 11,000 calls of volunteers to hospitalized patients). Preliminary qualitative results show that patients found high level of satisfaction receiving both these services.^15^

The third lesson was the importance of constantly supporting the CHUM community and communicating authentically and in real time. Psychological impact and infection rates were mitigated by this approach (no deaths among our employees and less than 1% infected). Multiple supportive actions and means were deployed to protect and support employees, such as the ones previously described, plus the opening of a daycare for children of staff, free parking, bonuses, and in-hospital COVID screening.

We are now in the second wave of the COVID-19 crisis. The major problem will be the lack of human resources. Hence, supporting people, not only at work but in their personal lives, will be essential. Enabling creativity in people and teams and prioritizing innovations that save frontline workers time while addressing their fear, stress, and anxieties will be key. We also need to continue enhancing and catalyzing the adaptiveness of our organization and amplifying innovation in our larger ecosystem by working with an interdisciplinary, cross-sectorial, and inclusive approach.
